# The metabolic landscape of ovarian cancer stem cells: how do they survive?

**DOI:** 10.3389/fonc.2025.1738742

**Published:** 2026-01-16

**Authors:** Jixue Tan, Lin Tang, Qian Zhang

**Affiliations:** 1 Department of Obstetrics and Gynecology, West China Second Hospital, Sichuan University, Chengdu, China; 2 Emergency Obstetrics and Gynecology Department, West China Second University Hospital, Sichuan University, Chengdu, China; 3 Department of Obstetrics and Gynecology, Key Laboratory of Birth Defects and Related Diseases of Women and Children of Ministry of Education (MOE), State Key Laboratory of Biotherapy, West China Second Hospital, Sichuan University and Collaborative Innovation Center, Chengdu, China

**Keywords:** cancer metabolism, cancer stem cells, chemoresistance, metabolic plasticity, metastasis, ovarian cancer

## Abstract

Ovarian cancer remains one of the most lethal malignancies of the female reproductive system, with its high mortality rate largely driven by chemotherapy resistance and disease recurrence. Ovarian cancer stem cells (OCSCs), a small subpopulation within ovarian tumors, are characterized by their capacity for self-renewal, differentiation, and tumorigenic growth. They are recognized as central drivers of tumor initiation, metastasis, drug resistance, and relapse. Mounting evidence in recent years has highlighted the pivotal role of metabolic reprogramming in sustaining OCSC stemness and therapeutic resistance. In this review, we reported the major metabolic pathways engaged by cancer stem cells (CSCs), including glucose metabolism (glycolysis, the tricarboxylic acid cycle, oxidative phosphorylation, and reactive oxygen species regulation), lipid metabolism, and amino acid metabolism. These pathways function to meet the bioenergetic and biosynthetic requirements of CSCs. Particular emphasis is placed on the metabolic plasticity of OCSCs, which can transform between a relatively inactive quiescent state and a highly proliferative active state. This adaptability allows OCSCs to respond dynamically to microenvironmental changes, facilitate ovarian cancer implantation and metastasis, and evade chemotherapeutic stress. We further analyze the molecular networks governing OCSC metabolism, including key signaling cascades and transcription factors. From a therapeutic perspective, we discuss the anti-diabetic drug metformin, which has demonstrated potential in targeting CSC metabolism in both preclinical models and clinical studies. Finally, we outline future research directions aimed at exploiting the metabolic vulnerabilities of OCSCs. We highlight that combination strategies targeting metabolism hold significant potential for overcoming treatment resistance and preventing ovarian cancer recurrence.

## Introduction

Cancer is one of the greatest threats to human health and continues to be a central challenge in biomedical research. In recent years, the tumor heterogeneity concept has attracted much attention. Today, tumors are understood as complex systems assembled from different cellular niches, among them, cancer stem cells (CSCs) are a subclass of cells with self-renewal property and multilineage differentiation potential (1). CSCs contribute to several adverse clinical outcomes. They promote metastasis, epithelial–mesenchymal transition (EMT) and resistance to radiotherapy and chemotherapy (2). Understanding the survival mechanisms of CSCs is therefore essential for unraveling the fundamental biology of cancer and developing novel therapeutic strategies (3).

Another outstanding feature of tumor cells is their characteristic metabolic reprogramming that underlies survival and malignant growth. Regardless of oxygen availability, tumor cells generally use glucose to generate ATP to support rapid tumor growth. This phenomenon is called Warburg effect (4). This strategy provided not only energy, but also diverted glycolytic intermediates for biosynthesis such as the pentose phosphate pathway and the serine pathway to provide precursors for the formation of nucleotides, lipids, and amino acids (5, 6). Through metabolic reprogramming, tumor cells enhance their adaptability to dynamic microenvironments and promote malignant proliferation (7, 8).

Initially, CSCs were considered to share similar metabolic features with the bulk of tumor cells. However, in recent years, CSCs were identified as containing unique metabolic programs. Some evidence suggested that CSCs are primarily reliant on oxidative phosphorylation (OXPHOS), whereas others showed the importance of glycolysis as a metabolic energy source in CSCs (7). In addition, CSCs have also shown distinct preferences for lipid metabolism and amino acid metabolism. Recent reviews have emphasized the importance of CSC metabolism in cancer initiation, progression, and metastasis and underscore its critical role as an area deserving more attention to systematic analysis (9, 10).

Ovarian cancer is the deadliest gynecological malignancy with a prognosis of less than 50% 5-year survival rate (11). It is highly prone to chemotherapy resistance and metastasis, imposing a substantial clinical burden. Given the essential role of CSCs in tumor progression, a deeper understanding of ovarian CSC (OCSC) metabolism is important for improving patient prognosis. Building on high-level studies published in the last 5 years, this review sums up the main metabolic pathways that CSCs participate in, the main steps and molecular mechanisms of the OCSC metabolic regulation and the emerging approaches targeting OCSCs, with the aim of revealing the role of metabolism in OCSC survival and its implication in OCSC therapy.

## Overview of CSCs

CSCs, also known as tumor-initiating cells (TICs), constitute a distinct tumor subpopulation defined by their capacity for self-renewal and multilineage differentiation. Accumulating evidence shows that these malignant traits of CSCs are closely correlated with CSC plasticity -- particularly their metabolic plasticity. This metabolic flexibility enables CSCs to adapt to diverse microenvironments (12). Therefore, targeting the CSC and controlling its plasticity is a new approach for therapeutic treatments.

Surface markers are widely employed for CSC identification. Researchers have described a variety of CSC markers, with some shared across tumor types. CD133, a transmembrane glycoprotein, is one of the best-established markers and has been identified in multiple solid tumors, including pancreatic carcinoma (13), breast cancer (14), liver cancer (15), ovarian cancer (16), oral squamous cell carcinoma (17), and melanoma (18). Aldehyde dehydrogenase (ALDH), detected through specialized fluorescence-based assays, is a functional CSC marker in breast, ovarian, bladder, head and neck, and lung cancers (19). ALDH activity contributes to reactive oxygen species (ROS) scavenging and maintenance of stem cell homeostasis (20). CD44, the predominant hyaluronic acid receptor, mediates adhesion-related signaling pathways and has been implicated in oral cancer, esophageal carcinoma, and colorectal cancer (17, 21, 22). In addition to surface proteins, core pluripotency transcription factors including SOX2, NANOG, and OCT4 also serve as intracellular markers for CSC identification (23–25).

**Figure 1:**
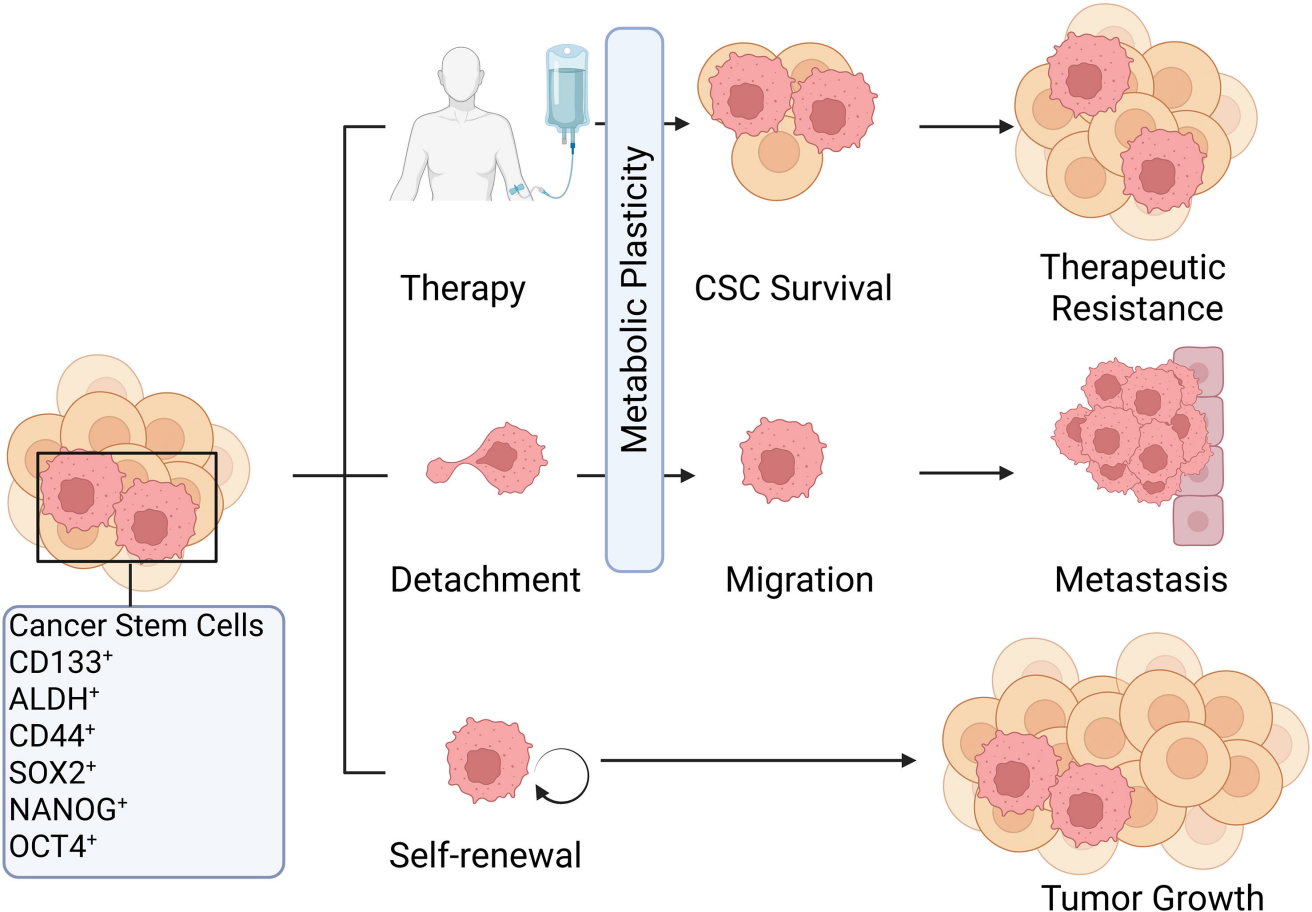
Surface markers and functional roles of CSCs in tumor progression. This figure depicts commonly used CSC surface markers, including CD44, CD133, and ALDH1, and outlines their critical roles in driving tumor growth, metastasis, and therapy resistance. The figure further highlights metabolic plasticity as a central mechanism enabling CSC-mediated metastasis and treatment resistance. #the figures in this article are Created with BioRender.com.

Although many studies have investigated surface markers of CSCs, these markers are not considered the gold standard for CSC identification. The definition of CSCs is fundamentally based on their functional properties, including self-renewal capacity and tumor-initiating potential. Accordingly, a range of functional assays and culture methods have been developed to isolate and expand CSCs. *In vitro*, researchers exploit CSC traits such as anchorage-independent growth and adaptation to serum-free culture by employing low-adhesion plates and serum-free media, thereby selectively inducing and enriching the formation of three-dimensional tumor spheres primarily composed of CSCs (26). *In vivo*, the remarkable tumorigenic capacity of CSCs has been proved through serial transplantation assays. This standardized protocol involves implanting tumor cells into immune-deficient animal models to establish primary tumors. Then isolating cells from these tumors, and re-implanting them into a secondary host (27). This cyclical process generates CSCs in a selective way that enriches the most tumor-initiating powerful sub-population of CSCs. Together, these methods for isolating, identifying, and characterizing CSCs have contributed greatly to research in this field. They further deepen our understanding about the molecular factors involved in the biology of CSCs, especially for OCSCs, and provide the solid groundwork for further studies. The surface markers and functional roles of CSCs in tumor progression is shown in **Figure 1**.

There has been a large amount of research focused on the molecular dynamics active within OCSCs in recent years, highlighting the key roles that diverse regulatory processes and complex interactions play in shaping OCSC formation. Stemness maintenance within OCSCs is coordinated and regulated by multiple evolutionarily conserved signaling pathways, including the Wnt, NF-κB, Notch, Hedgehog, JAK–STAT, and PI3K/AKT/mTOR signaling axes (23). Moreover, intracellular mechanisms such as epigenetic reprogramming and metabolic plasticity cooperate with extracellular elements of the tumor microenvironment. These include hypoxic conditions and interactions with tumor-associated fibroblasts (CAFs), immune cells, adipocytes, and other stromal elements (28–31). Collectively, these factors identify a multidimensional regulatory network that strongly shapes OCSC self-renewal and therapy-resistant phenotypes. For information on the molecular mechanisms and markers for OCSCs, please refer to our previous review (32).

## Metabolic activity in CSCs

**Figure 2:**
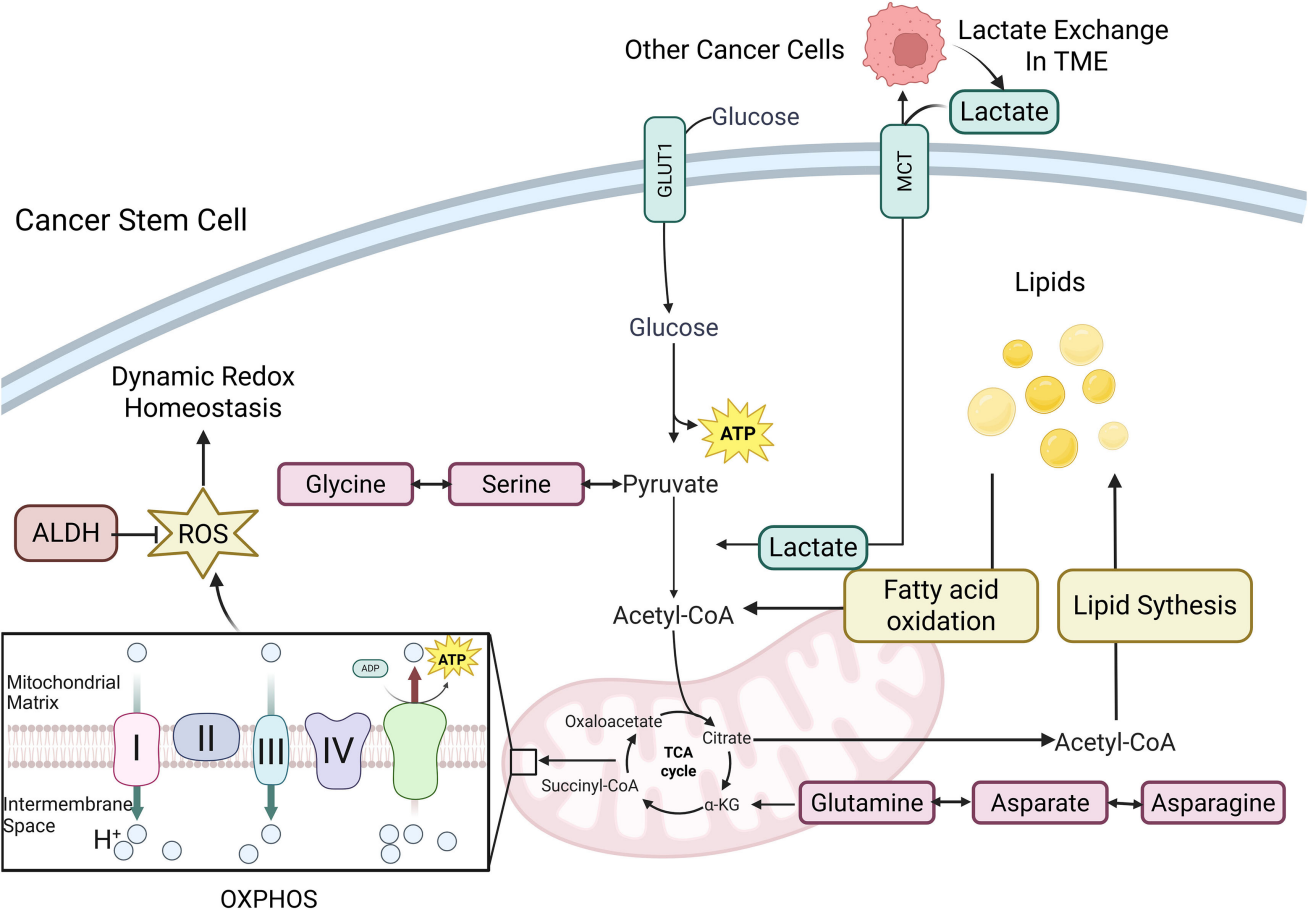
Interconnected metabolic networks governing CSC metabolism. This figure summarizes the major metabolic pathways in CSCs, including glycolysis, oxidative phosphorylation (OXPHOS), lipid metabolism, and amino acid metabolism. It illustrates how these pathways are highly interconnected rather than isolated, with key metabolites shuttling between them to establish a flexible and resilient metabolic network. Notably, OXPHOS serves as a principal source of mitochondrial reactive oxygen species (ROS), which contribute to dynamic redox homeostasis. In parallel, the glycolytic end-product lactate is exported from CSCs via monocarboxylate transporters (MCTs), thereby facilitating lactate shuttling within the tumor microenvironment (TME). #the figures in this article are Created with BioRender.com.

CSCs display significant metabolic plasticity, including glucose, fatty acid, and amino acid metabolism to sustain stemness and survival. In the case of OCSCs, the metabolic adaptability of OCSCs is characterized by distinct, interconnected programs. These distinct metabolic programs help provide chemoresistance and drive recurrence in them. The interconnected metabolic networks are shown in **Figure 2**.

### Glucose metabolism

Glucose is an important metabolic substrate for tumor cells. Traditionally, tumor cells are thought to use glycolysis, or the Warburg effect, as the main pathway to produce energy. New data demonstrate that CSCs do not follow this; that is, they do not exclusively depend on glycolysis, and aerobic respiration has a significant role as well (33). This section discusses the contributions of aerobic glycolysis and aerobic respiration to CSC biology.

#### Aerobic glycolysis

Glycolysis represents a fundamental metabolic pathway through which glucose is degraded in the cytoplasm to support energy metabolism without needing oxygen. In glycolysis, 1 molecule of glucose yields 2 molecules of pyruvate, 2 ATP, and 2 NADH. Glycolysis is involved in CSC stemness maintenance, functioning as a source of chemoresistance, and driving malignant progression (34, 35). Glycolysis regulation can be mediated by a variety of mechanisms. For example, in oral squamous cell carcinoma, Epstein–Barr virus-infection related glycolysis proteins, like LDHA, GLUT1 and PDK1, incite the production of lactate and strengthen the cancer cell stemness (17). In glioblastoma CSCs, transcription factor YY1 upregulates expression of GNG5 to activate Wnt/β-catenin signaling, to facilitate rapid glucose uptake and glycolytic flux, and thus boost the proliferation, invasion, and treatment resistance of CSCs (36). Similarly, in glioblastoma, aryl hydrocarbon receptor nuclear translocator (ARNT) conserves a high level of glycolytic activity by stabilizing the p38α-MAPK pathway, which protects glioblastoma CSCs from temozolomide cytotoxicity. Targeting ARNT destabilizes p38α, reduces the expression of glycolytic enzymes, and sensitizes glioblastoma cells to chemotherapy (37). In head and neck squamous cell carcinoma, the tyrosine kinase EGF phosphorylates PKM2 and activates PKM2, activating this glycolysis-associated enzyme and significantly enhancing CSC stemness (38).

Conversely, suppression of glycolysis is often associated with a loss of stemness. For example, CD44 knockout in the glioma cell downregulates glycolysis-related proteins and thus inhibits stemness of the CSC cell and cell-cycle progression, suggesting that metabolic collapse may be related to loss of CSC properties (39). Mamouni et al. found in bladder CSCs, scaffold protein ARRB1 drives tumor glycolysis by inhibiting the mitochondrial pyruvate carrier MPC1. Meanwhile, ARRB1 promote GLUT1 expression and glucose uptake. Those glucose-induced processes promote breast CSC stemness (40).

In summary, Glycolysis provides the metabolic basis for CSC stemness, chemoresistance and metastasis. Targeting key regulators of glycolysis is therapeutically desirable to target CSC.

#### Aerobic respiration: TCA cycle and OXPHOS

Although the Warburg effect of glycolysis emphasizes glycolysis as the predominant metabolic pathway in tumor cells, recent studies emphasize the vital roles of the tricarboxylic acid (TCA) cycle and OXPHOS in the metabolism of CSCs (41, 42).

TCA cycle takes place in the mitochondrial matrix and forms the largest intersection in aerobic respiration. It not only generates ATP but also provides intermediates for proteins, lipids and nucleic acids for cell synthesis. The TCA cycle generates reducing equivalents, including NADH, FADH2 to fuel OXPHOS (43). Prostate CSCs metabolism relies on the TCA cycle coupled with OXPHOS. In prostate CSCs, activation of mitochondrial aconitase (m-ACO2) reduced intracellular zinc levels. By repressing the zinc transporter ZIP1 with the nuclear receptor ERRα, cellular uptake of zinc was diminished, causing the activation of ACO2 to fulfill the TCA cycle. Restoring zinc levels with the zinc ionophore clioquinol substantially suppressed CSC growth *in vitro* and tumorigenicity *in vivo*, suggesting zinc supplementation as a therapeutic approach (44). In acute myeloid leukemia CSCs, the compound ONC213 inhibited α-ketoglutarate dehydrogenase (α-KGDH), an enzyme of the TCA cycle. This inhibition leads to α-KGDH accumulation, induces mitochondrial stress, and concurrently reduces translation of the anti-apoptotic protein MCL1. These combined effects suppress OXPHOS and effectively eliminate CSCs. Importantly, ONC213 demonstrated efficacy in relapsed patient-derived xenograft models while sparing normal hematopoietic cells (45). Taken together, all these findings demonstrate how the TCA cycle play act as a metabolite hub in CSCs, providing energy and works as a mitochondrial signal center that regulates stemness and anti-tumor drug resistance.

OXPHOS is a critical metabolic pathway for cellular energy production. OXPHOS takes place on the inner mitochondrial membrane in which the electron transport chain produces energy by generating ATP from nutrient oxidation. OXPHOS is thought to be a key factor for maintaining stemness and therapy resistance in CSCs. Liu et al. demonstrated that liver CSCs exhibit significantly elevated OXPHOS activity compared with differentiated hepatoma cells. Silencing OXPHOS components suppressed stemness markers by inhibiting mitochondrial fission (46). Similarly, in acute myeloid leukemia, a specific subtype of MLL/AF9 fusion-driven CSCs displayed threefold higher OXPHOS activity than normal hematopoietic stem cells, creating a metabolic vulnerability to metformin (47). Colorectal CSCs also upregulate OXPHOS to evade the cytotoxic effects of 5-fluorouracil (5-FU) (48). In prostate CSCs, estrogen-related receptor α (ERRα) regulates mitochondrial genes and zinc transport to sustain tumorigenicity (44). Loss of the tumor suppressor MYBBP1A enhances OXPHOS and metabolic plasticity in colorectal CSCs, thereby promoting tumorigenicity (49). In non-small cell lung cancer, inhibition of LDHB disrupts mitochondrial membrane potential, induces mtDNA damage, and selectively kills CSCs (50). In most contexts, activation of OXPHOS is correlated with CSC proliferation and maintenance.

Conversely, inhibition of OXPHOS can selectively eradicate therapy-resistant CSCs. For example, stem cells of chemoresistant chronic myeloid leukemia (CML) rely on OXPHOS. Inhibition of this pathway suppresses colony formation in patient-derived CD34+ CML cells, and combining the OXPHOS inhibitor NK-128 with imatinib significantly improves therapeutic outcomes in mouse models (51).

#### Reactive oxygen species

ROS, produced as endogenous byproducts of oxidative metabolism, primarily originate from electron leakage at mitochondrial respiratory chain complexes I and III during OXPHOS. Traditionally, these species are regarded as toxins to the cell, but recent work reveals that at physiological concentrations, ROS-producing and ROS-scavenging systems forms tightly regulated network that mediates redox homeostasis. Disturbing this balance whether by inducing excessive production or depletion of ROS produces profound changes in intracellular state (52). Interestingly, ROS can function as a double-edged sword for CSCs.

ROS accumulation inhibits CSC viability in many cases. For instance, drug-resistant pancreatic CSCs have low levels of ROS and high OXPHOS to avoid gemcitabine elimination (53). The powerful CSC marker enzyme ALDH maintains CSCs’ stemness by detoxifying aldehydes and inhibiting ROS (54). Guo et al. further demonstrated that the disulfiram/copper therapy can efficiently eliminate the ALDH positive OCSCs by inhibiting ALDH activity and causing a ROS burst (20). Similarly, through RNA sequencing and high-throughput screening, Harrington et al. found that disulfiram and salinomycin induce oxidative stress via the OXPHOS/ROS axis, markedly downregulating OCSC stemness markers (ALDH/CD133), enhancing carboplatin cytotoxicity, and suppressing recurrence *in vivo* (55).

However, ROS can also induce CSC stemness under certain conditions. In glutamine-deprived tumor microenvironments, ROS accumulation upregulates the MAPK–ERK1/2 signaling pathway and causes mitochondrial fragmentation, promoting expansion of CD44+/CD117+ OCSC population (56). This paradox demonstrates a key principle: ROS effects on CSCs are highly context-dependent, which is determined by microenvironmental pressure, cell type and all downstream interactions.

### Lipid metabolism

Many tumor cells use lipid metabolism to sustain relentless growth and spread. Fatty acid oxidation (FAO) supplies metabolic energy to sustain CSC proliferation. Lipids-derived molecules, such as cholestenones and phosphates, also serve as signaling molecules that regulate CSC survival and stemness.

FAO is particularly important for CSC survival and progression. In a Barrett’s esophagus mouse model, a high-fat diet enhanced palmitate uptake, upregulated the fatty acid oxidation rate-limiting enzyme CPT1A, and increased mitochondrial fatty acid oxidation. These metabolic changes drove epithelial cell proliferation, upregulated stemness markers, and activated IL-8 inflammatory signaling. This established a vicious “metabolism-inflammation-stemness” cycle that promoted esophageal adenocarcinoma progression (57). In glioblastoma, the SREBP1 inhibitor TAK901 suppressed lipid synthesis, resulting in reduction of CSC and tumor cell malignancy (58). In triple-negative breast cancer (TNBC), downregulation of the stem cell marker CD24 reprogrammed mitochondrial metabolism to enhance FAO, through the PPARα-NF-κB-CPT1A axis (59).

Beyond energy supply, lipid metabolism also supports biosynthesis and regulates CSC signaling. In breast cancer, lysosphingolipid sphingosine-1-phosphate (LPA) sustains CSC phenotypes by activating LPAR3, which opens TRPC3 calcium channels. This elevates intracellular Ca^2+^ levels, activates NFAT, and drives IL-8 secretion, collectively supporting breast CSC stemness (60). In high-grade serous ovarian cancer, squalene epoxidase (SQLE), a key cholesterol metabolism enzyme, was found to be upregulated fourfold in patient samples and correlated with poor prognosis. SQLE overexpression promoted CSC traits *in vitro* and *in vivo*, with metabolomic analyses showing dysregulated cholesterol and glutathione metabolism (61). In chronic myelogenous leukemia stem cells, lysophospholipids function as second messengers essential for stemness. The enzyme Gdpd3 regulates lysophospholipid metabolism, simultaneously inhibiting the proliferative AKT/mTORC1 pathway while activating FOXO and β-catenin to maintain stemness. This dual regulatory mechanism enables CML stem cells to persist long-term in a quiescent state and develop resistance to tyrosine kinase inhibitors (TKIs) (62).

In summary, FAO fuels the energy demands of CSCs during metastasis and drug resistance while also sustaining their stemness. Therefore, targeting this central survival pathway is a rational strategy for eradicating CSCs in combination with conventional treatments.

### Amino acid metabolism

Proteins are fundamental building blocks of life, and amino acids provide indispensable nutrients essential for biological processes. The 20 amino acids in humans can be classified into three groups: (i) essential amino acids, which cannot be synthesized endogenously and must be obtained from diet; (ii) semi-essential amino acids, such as arginine and histidine, which require dietary intake or precursor availability (e.g., methionine and phenylalanine); and (iii) non-essential amino acids, which can be synthesized internally (63). Crucially, each amino acid, whether essential or non-essential, exerts distinct physiological functions, and influences CSC metabolism.

Certain amino acids directly contribute to cellular survival and energy metabolism. Methionine and glutamine function as nutritional substrates for CSC survival, while tyrosine metabolism provides energizing fuel for CSC proliferation (63, 64). In addition, several amino acids, including glutamine, glutamate, aspartate, asparagine, and glycine, all feed into the TCA cycle (63, 65, 66). Beyond their metabolic roles, the amino acid derivatives function as signaling molecules as well. For example, indoleamine 2,3-dioxygenase 1 can be catalyzed to tryptophan metabolism and thus generate kynurenine, forming a positive feedback loop between the kynurenine metabolism and the Notch pathway. The positive interaction enhances radioresistance in cervical CSCs (67).

Furthermore, CSCs indirectly remodel their metabolic profiles through indirect mechanisms, such as ROS modulation. For instance, in OCSCs, glutamate shortage contributes to an ROS accumulation in OCSCs, leading to the activation of the MAPK-ERK1/2 pathway. This cascade facilitates DRP1-induced mitochondrial fragmentation, which ultimately enlarges the number of OCSCs in mice (56). Collectively, these results illustrate how amino acid regulates CSC function both through direct metabolic inputs and through signaling-mediated regulation of stemness phenotypes.

## OCSC metabolic plasticity

Recent research has demonstrated striking metabolic heterogeneity of CSCs, characterized by paradoxical states in which both high-OXPHOS/high-ROS and low-OXPHOS/low-ROS profiles can sustain stemness (16, 52, 56). Such heterogeneity reflects transitions between stem cell quiescence and activated stem cell states (68).

**Figure 3:**
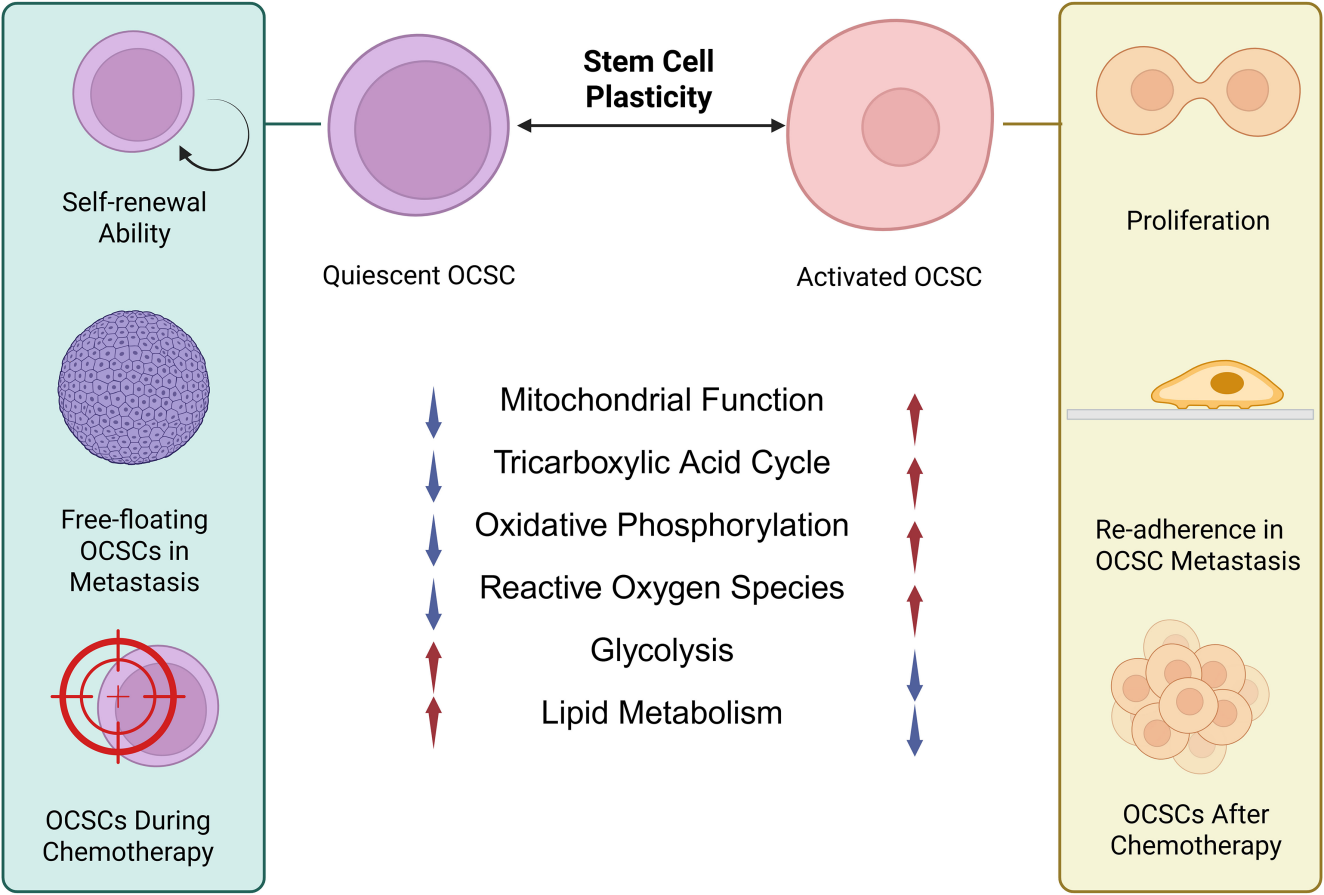
Functional duality of quiescent and active OCSCs. This figure illustrates that OCSCs exist in two interconvertible states, each characterized by distinct metabolic signatures and functional roles. Quiescent OCSCs are essential for long-term self-renewal, survival during the free-floating stage of metastasis, and persistence under therapeutic stress. In contrast, active OCSCs are proliferative, driving metastatic re-adhesion and post-treatment relapse. This ability to transition between states exemplifies OCSC metabolic plasticity, enabling adaptation to environmental pressures and coordination of complementary functions in tumor progression. #the figures in this article are Created with BioRender.com.

Quiescent stem cells, in oxygen-restricted niches, are generally characterized by low activity, low content of mitochondria and reliance on glycolysis and lipid metabolism. When activated by microenvironment cues or growth factors, these cells toward proliferative self-renewal and differentiation, accompanied by decreased glycolytic dependence and increased mitochondrial OXPHOS (65). These observations show metabolic plasticity, which is a defining CSC characteristic that describes the ability to dynamically reprogram metabolic pathways in response to environmental stressors (69). The active and quiescent duality of the two populations of OCSCs is shown in **Figure 3**.

Plasticity functions as a common regulatory mechanism that drives CSC metastasis, drug resistance and relapse (70). All proliferative states of CSCs show enhanced OXPHOS, higher lipid metabolism and increased oxygen consumption (56). Metastasis requires many metabolic adaptations (70). Drug resistance arises from distinct metabolic adaptations in different CSC subpopulations. These subpopulations may rely on either highly active OXPHOS or hypoxia-driven metabolic pathways, which are engaged in response to specific therapeutic pressures (71, 72).

During ovarian cancer metastasis—which requires “detachment” followed by “re-adhesion”—the OCSCs exhibit a distinct type of metabolic reprogramming (73). “Detachment” here is driven mostly by EMT and is strongly correlated with high glycolytic activity. For example, overexpression of hexokinase 2 (HK2) increases glycolytic efficiency in OCSCs and increases lactate production, acidifies the tumor microenvironment, and thereby promotes migration, invasion, and metastatic niche formation *in vivo* (72). By contrast, OCSCs treated with the glycolytic inhibitor lonidamine prevent the progression of EMT and stemness *in vitro* (74). In contrast, during re-adhesion phase, OCSCs adopt a distinct metabolic profile. Indeed, simulation experiments by Compton et al. concluded that free-floating OCSC spheroids, upon reattaching at secondary sites, shift their metabolic profile from glutamine dependency to metabolism by glucose-mediated OXPHOS, while maintaining steady FAO activity (75). Thus, OCSCs manifest context-dependent metabolic states, such that the detachment phase resembles quiescent stem cells (glycolytic, lipid-dependent, low oxygen consumption), whereas the re-adhesion phase resembles activated stem cells (glucose-fueled OXPHOS). Such adaptability implies metabolic plasticity required for the OCSC survival and metastatic colonization.

Drug resistance of OCSC is also driven by metabolic plasticity. During chemotherapy, OCSCs respond to the stress by reprogramming glucose and lipid metabolism. In ovarian cancer, drug-resistance is marked by increased lipid metabolism. Artibani et al. perform multi-region transcriptomic analyses on paired pre- and post-chemotherapy biopsy samples from 17 patients and detected an adipocyte-like transcriptional signature of minimal residual disease (MRD) cells that overlapped with OCSC markers (76). Further *in vitro* studies confirmed that MRD-like cells depend on FAO for survival and chemoresistance (76). For example, Huang et al. showed that accumulation of periostin in the microenvironment of recurrent ovarian cancer upregulates fatty acid synthase (FASN) in OCSCs (77). Moreover, aberrant activation of fatty acid desaturases SCD1 and FADS2 in ovarian cancer peritoneal metastases enhance lipid metabolism, facilitating tumor invasion. Subsequently pharmacological inhibition or genetic silencing of SCD1/FADS2 led to delay of tumor growth, suppressed CSC formation, and reversal of platinum resistance. Pre-clinical trials also demonstrated that combining SCD1/FADS2 inhibitors together with cisplatin to blocked tumor dissemination, suggesting a promising therapeutic strategy for peritoneal metastasis in ovarian cancer (78).

Role of glucose metabolism in chemoresistance is less clear. Some studies give a dominant role of aerobic metabolism. For example, CPI-613, a TCA cycle inhibitor, prevents enrichment of OCSCs after chemotherapy (79). Sriramkumar et al. have shown that platinum-based chemotherapy activates SIRT1, which promote OXPHOS in OCSCs, leading to ALDH^+^ stem cell expansion, chemoresistance, and recurrence. Combining OXPHOS inhibitors with SIRT1 inhibitors synergistically promoted prolonged survival of tumor-bearing mice. In contrast, other studies suggest that increase in glycolysis contributes to chemoresistance of OCSCs (80). For example, overexpression of HK2 increases lactate production, acids the microenvironment and induces upregulation of the FAK/ERK1/2 pathway, conferring resistance (69).

There is also evidence of synergistic contributions from both glycolysis and aerobic metabolism. Wang et al. documented that BAG5-mediated resistance is associated with concurrent upregulation of both pathways (71). Taken together, these findings highlight the central role of metabolic plasticity in the treatment resistance of OCSCs. Similar to metastasis, this adaptability allows OCSCs to survive therapeutic stress, which reinforces the need to understand how microenvironmental changes will regulate metabolite reprogramming of OCSCs for the development of powerful targeted therapies.

### Regulation of OCSC metabolism

Multiple factors regulate the metabolism of OCSCs. **Table 1** lists major molecules identified in the last 5 years. Among them, the AKT/mTOR pathway is among the most dominant regulators. This pathway is frequently mutated in tumors and controls several cancer cell processes, including cell cycle, survival, metabolism, motility, and genomic stability (78). More specifically in OCSCs, activation of AKT/mTOR promotes chemotherapy resistance, tumorigenesis, EMT and stemness maintenance (85–89). In breast CSCs, α-lipoic acid (LA) can inhibit the activity of PI3K to further inactivate AKT/mTOR downstream signal. This inhibition inhibits glycolysis and amino acid metabolism, ultimately inducing apoptosis and impairing self-renewal capacity of breast cancer CSCs (90).

**Table 1:** Molecules that regulate OCSC metabolism.

Regulatory molecules	Metabolic pathway	Regulatory mechanism	Functional outcome
MNRR1 (81)	OXPHOS	Promotes cell survival through Bcl-xL binding and enhances respiratory capacity via interaction with COX IV	Improves tumor growth and metastasis
Periostin (77)	Lipid metabolism	Stimulates FASN and activates the AKT signaling pathway	Promotes stemness, chemoresistance and recurrence
DRP1 (56)	Glutamine metabolism	Glutamine deficiency results in DRP1 phosphorylation, causing mitochondrial fragmentation	Enhances the number of OCSCs
HK2 (72)	glycolysis	HK2 enhances stemness properties via the FAK/ERK1/2/NANOG/SOX9 signaling pathways and promotes glycolysis	Promotes stemness and metastasis
LAMP2A (82)	Fructose metabolism	Fructose metabolism can promote LAMP2A-mediated chaperone-mediated autophagy, enhancing stemness marker expression while also promoting ferroptosis	Both inhibits and enhances stemness
SIRT1 (80)	OXHPOS	Platinum treatment induces the upregulation of SIRT1, enhances deacetylase activity, and causes mitochondrion and OXPHOS activation	Promotes stem cell enrichment, chemoresistance
BAG5 (71)	Glycolysis and OXPHOS	The Bcl6/BAG5/Rictor-mTORC2 axis mediates metabolic reprogramming, driving enhanced glycolysis and OXPHOS	Promotes cisplatin resistance
SCD1/FADS2 (78)	Lipid metabolism	SCD1 and FADS2 upregulation in ascites-driven ovarian cancer promotes unsaturated fatty acid synthesis, suppressing ferroptosis via the GPX4-GSH axis	Promotes cisplatin resistance
FABP4 (83)	Lipid metabolism	Adipocyte-induced FABP4 upregulation in ovarian cancer cells enhances lipid metabolism	Promotes metastasis
Frizzled-7 (84)	Glutathione metabolism	FZD7 activates the Tp63-glutathione/GPX4 axis to protect OCSCs against oxidative stress, while GPX4 inhibition induces ferroptosis in OCSCs	Prevents ferroptosis

Conversely, in colon cancer CSCs, enhanced PI3K catalytic activity increases the activity of the mitochondrial state (91). Collectively, these observations place the AKT/mTOR axis at the epicenter of CSC metabolic control, while PI3K acts as a master intracellular enabler of metabolism and the change from quiescence to activation plasiticity of CSCs. **Table 1** Molecules that Regulate OCSC Metabolism.

### Efforts to disrupt CSC metabolism in the clinic

Currently, several drugs targeting tumor cell metabolism are already available in clinical practice. Within the context of CSC metabolism, metformin is a commonly prescribed antidiabetic drug with a well-established safety profile that has been extensively investigated for its anticancer potential. Robust clinical evidence indicates that metformin can reduce cancer incidence and improve prognosis (92). Although its precise mechanism of action remains incompletely defined, metformin is known to inhibit the AKT/mTOR pathway and block OXPHOS, thereby forcing CSCs to shift from an OXPHOS-dependent phenotype to anaerobic glycolysis. This metabolic switch suppresses the proliferation and metastatic capacity of activated CSCs (93). For example, metformin markedly inhibits EMT induction, which is a hallmark of CSC activation. In nude mouse models, metformin reverses the mesenchymal and EMT-like properties of intrahepatic cholangiocarcinoma stem cells by activating the AMPK-FOXO3 signaling axis, suggesting significant therapeutic potential (94). Metformin also directly impairs mitochondrial function in CSCs, and its combination with chemotherapeutic agents has been shown to markedly enhance efficacy. *In vitro*, treatment of endometrial CSCs with metformin reduced mitochondrial membrane potential and increased extracellular lactate accumulation, underscoring its ability to impair CSC mitochondrial bioenergetics (95). Collectively, these findings indicate that metformin suppresses activated CSCs by targeting both signaling and metabolic pathways.

In addition to targeting activated CSCs, metformin increases the metabolic vulnerability of quiescent CSCs, rendering them more susceptible to chemotherapy. In endometrial carcinoma CSCs, for instance, metformin significantly impaired mitochondrial homeostasis by reducing membrane potential and increasing extracellular lactate. This reprogramming effect synergized with the MYC signaling inhibitor KJ-Pyr-9 and the chemotherapeutic drug carboplatin to eliminate endometrial CSCs, as evidenced by the downregulation of stemness markers (CD133/CD44) and EMT markers (Twist, Snail, Slug) (95). Similarly, in chemoresistant hepatocellular carcinoma CSCs, quiescent cells rely on glutamine metabolism to generate ATP, which supports P-glycoprotein (P-gp)-mediated drug efflux. Exploiting this vulnerability, combined treatment with doxorubicin and metformin under glutamine-free conditions suppressed P-gp function and restored sensitivity to doxorubicin (64).

It has been further demonstrated in clinical settings that metformin exerts effects on OCSCs. A phase II trial involving 38 non-diabetic patients with advanced epithelial ovarian cancer demonstrated that a metformin-containing neoadjuvant/adjuvant regimen significantly improved outcomes, including prolonged progression-free survival and reduced recurrence. Metformin treatment reduced ALDH+CD133+ OCSCs by 2.4-fold and enhanced ex vivo cisplatin sensitivity. Moreover, metformin altered the methylation profile of carcinoma-associated mesenchymal stem cells, thereby disrupting their role in mediating chemoresistance (96). In addition, ongoing clinical trials are investigating the use of metformin against CSCs in colorectal cancer, gynecological malignancies (including ovarian, fallopian tube, and primary peritoneal cancers), and non-small cell lung cancer, though results are not yet available (97–99). Together, these findings support further clinical evaluation of metformin as an OCSC-targeting adjuvant therapy. A deeper understanding of OCSC metabolism will likely facilitate the identification of new therapeutic vulnerabilities.

## Prospects and challenges

CSCs provide an essential perspective for understanding disease intractability through their strong capacity for metabolic reprogramming. At the same time, they open promising avenues for therapeutic innovation. In ovarian cancer, however, research specifically focused on OCSC metabolism remains limited, offering a fertile ground for future exploration.

Despite this therapeutic potential, OCSCs research faces substantial challenges, primarily due to the remarkable metabolic plasticity of these cells. Specific surface markers for OCSCs remain poorly defined, and the heterogeneity of OCSC subpopulations marked by different surface antigens, as well as the possibility that these markers change dynamically with cell state, remains unresolved. Moreover, metabolic data derived from *in vitro* experiments often fail to capture the complexity of the *in vivo* tumor microenvironment. Constructing reliable *in vivo* models also remains difficult, and accurately tracking, isolating, and eradicating OCSCs continues to present significant obstacles. These limitations restrict the depth of mechanistic insights and impede clinical translation. Moreover, OCSCs exhibit extensive adaptability across nearly all major metabolic pathways. This poses significant challenges for designing metabolism-targeting interventions. However, it also presents a major opportunity: by disrupting this adaptive network, we may effectively inhibit and eradicate OCSCs.

Looking forward, these challenges also highlight potential directions for advancement. The rapid development of metabolomics and single-cell multi-omics, including the integration of scRNA-seq with metabolic flux analyses, will enable fine-grained profiling of OCSC metabolic signatures and nutrient utilization patterns. This integrated approach will improve our understanding of OCSC heterogeneity, stemness hierarchy, metastatic potential, and therapeutic response by revealing dynamic metabolic adaptations. Similarly, the increasing use of lineage-tracing technologies *in vitro* and *in vivo* will offer unprecedented opportunities to dissect OCSC dynamics in real time. Advances in experimental modeling also hold promise, with 3D organoid co-cultures, patient-derived models, organ-on-a-chip systems, and genetically engineered mouse models enabling more physiologically relevant studies. Optimized patient-derived xenograft models, combined with advanced metabolic imaging and omics approaches, may further increase the predictive value of preclinical research. From a therapeutic perspective, repurposing existing metabolism-targeting drugs such as metformin is a practical starting point, while novel drug delivery technologies, including nanoparticles, offer opportunities to improve specificity and efficacy against OCSCs.

In conclusion, targeting OCSC metabolism represents a highly promising direction in cancer therapy. Overcoming ovarian cancer recurrence and treatment resistance will require continued mechanistic investigation, innovative technologies, and optimized preclinical models. With increased research investment and interdisciplinary collaboration, significant breakthroughs are achievable, paving the way for more effective treatments against ovarian cancer.
